# Metal Nanoparticle-Decorated Two-Dimensional Molybdenum Sulfide for Plasmonic-Enhanced Polymer Photovoltaic Devices

**DOI:** 10.3390/ma8085252

**Published:** 2015-08-21

**Authors:** Ming-Kai Chuang, Shun-Shing Yang, Fang-Chung Chen

**Affiliations:** Department of Photonics, National Chiao Tung University, Hsinchu 30010, Taiwan; E-Mails: secret0622.di97g@g2.nctu.edu.tw (M.-K.C.); johnnye95@gmail.com (S.-S.Y.)

**Keywords:** nanoparticle, molybdenum sulfide, plasmonic, polymer, solar cells

## Abstract

Atomically thin two-dimensional (2D) transition metal dichalcogenides have also attracted immense interest because they exhibit appealing electronic, optical and mechanical properties. In this work, we prepared gold nanoparticle-decorated molybdenum sulfide (AuNP@MoS_2_) through a simple spontaneous redox reaction. Transmission electron microscopy, UV-Vis spectroscopy, and Raman spectroscopy were used to characterize the properties of the AuNP@MoS_2_ nanomaterials. Then we employed such nanocomposites as the cathode buffer layers of organic photovoltaic devices (OPVs) to trigger surface plasmonic resonance, leading to noticeable enhancements in overall device efficiencies. We attribute the primary origin of the improvement in device performance to local field enhancement induced by the effects of localized surface plasmonic resonance. Our results suggest that the metal nanoparticle-decorated two-dimensional materials appear to have great potential for use in high-performance OPVs.

## 1. Introduction

Organic photovoltaic devices (OPVs) have received a great deal of attention because they feature many advantageous properties, including light weight, low cost, mechanical flexibility and short energy payback time [1,2,3]. To date, the power conversion efficiencies (PCEs) of the single-junction devices have broken through 10% [4]. Moreover, the internal quantum efficiency (IQE) of state-of-art OPVs can approach 100%, meaning that nearly every absorbed photon can be converted to charge carriers and that all the carriers are collected at the electrodes [5]. Because the overall quantum efficiency is governed by the IQE and absorption efficiency, efficient light absorption in OPVs is still critical for further improving the PCEs. One approach for increasing the absorption efficiency is to develop light trapping techniques [6,7,8,9,10,11,12]. For example, using optical spacers [7,8] and photonic crystals [9] have been recently proposed. Among the light-trapping schemes, incorporation of metal nanostructures, which can trigger surface plasmons (SPs), have been proved to be a promising way for increasing the light harvesting ability of OPVs [10,11,12,13,14,15,16,17,18,19,20,21,22,23]. As metal nanoparticles (NPs) can be readily synthesized and incorporated into the devices via simple solution processes, they have become the most widely used plasmonic nanostructures for enhancing the PCEs of OPVs [11,12,13].

Atomically thin two-dimensional (2D) transition metal dichalcogenides (TMDs) have also attracted immense interest because they exhibit appealing electronic, optical and mechanical properties [24,25,26,27,28,29]. In particular, the TMDs that having direct band gaps, such as MoS_2_ and WS_2_, have been employed in many applications [24,25], including field effect transistors [26], photodetectors [27], light-emitting devices [28] and sensors [29]. Further, these TMDs have been incorporated into OPVs as interfacial buffer layers for improving their device stability and/or efficiencies [30,31,32,33,34]. More interestingly, the work-function of the TMD interfacial layers can be modulated by *p*- or *n*-doping treatments [30]. Therefore, 2D TMDs can be considered as promising building blocks for preparing materials exhibiting various functionalities. Recently, Yang *et al.* prepared a hole transport layer composed of ultrathin 2D MoS_2_ nanosheets decorated with Au NPs for triggering the plasmonic effects in OPVs [35]. From both simulation and experimental results, they have shown that the nanocomposites can utilize the plasmonic near-field more efficiently, particularly along the horizontal direction, thereby leading to apparent efficiency improvement.

In this work, we have prepared MoS_2_ nanosheets using a very simple, greener liquid phase exfoliation method [36]. A surfactant was added into the suspension of bulk MoS_2_, and, thereby, a stable aqueous dispersion of exfoliated MoS_2_ sheets could be obtained after sonication. The resulting nanosheets were further decorated with Au NPs through a spontaneous redox reaction with hexachloroauric acid, resulting in a novel nanostructure of Au NP-decorated MoS_2_ (AuNP@MoS_2_) nanocomposites [37]. Note that no additional reducing agent was required to reduce the Au ions. The as–synthesized AuNP@MoS_2_ could be readily incorporated into the OPVs as an interfacial buffer layer between the active layer and the electrodes. We have found that the Au NPs anchored on the MoS_2_ nanosheets induced the SR effects, which could effectively improve the device performance.

## 2. Results and Discussion

### 2.1. Synthesis and Characteristics of Au NP-Decorated MoS_2_ Nanocomposites

To prepare the AuNP@MoS_2_ nanocomposites, bulk MoS_2_ was exfoliated through a liquid phase method [36]. A triblock copolymer, poly(ethylene glycol)-*block*-poly(propylene glycol)-*block*- poly(ethylene glycol) (Pluronic P123) was added into an aqueous suspension of bulk MoS_2_. It behaved as a surfactant to reduce and maintain the surface tension of the aqueous phase for efficient exfoliation [36]. As a result, a stable dispersion of MoS_2_ nanosheets could be obtained after sonication. Then, a solution of HAuCl_4_ that was dissolved in de-ionized (DI) water was mixed with the resulting MoS_2_ solution. We found that Au ions were spontaneously reduced and anchored on the surface of exfoliated nanosheets. No additional reducing agent was required in this spontaneous reaction [37].

**Figure 1:**
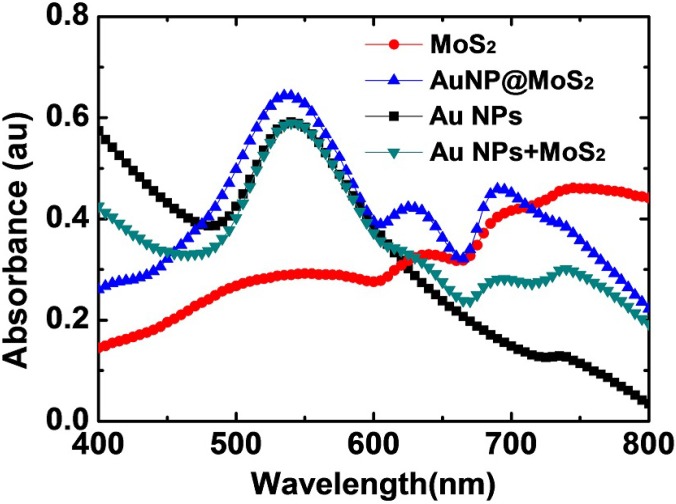
Absorption spectra of the nanocomposites dispersed in aqueous solutions, including Au NP-decorated MoS_2_ (AuNP@MoS_2_), Au nanoparticles (Au NPs), the MoS_2_ nanosheets (MoS_2_) and the mixture of the Au NPs and MoS_2_ nanosheets (Au NPs + MoS_2_).

Figure 1 displays the absorption spectra of the MoS_2_ nanosheets suspended in water; several features of the spectrum are similar to those reported previously [38]. First, two excitonic peaks at 690 and 645 nm, which are termed A and B excitons, respectively, could be observed. They are related to the interband excitonic transitions at the K point of the Brillouin zone for the nanosheets with large lateral dimensions. The energy difference between the two excitonic peaks is due to the effect of spin–orbital coupling of the valence band [36,39]. Second, we also clearly observed one more peak at 745 nm, which was primarily due to scattering [38]. Such scattering effects could be resulted from the highly anisotropic structure, poor dispersion of the nanosheets, and damaged surfaces [39]. Note that the much larger scattering cross section of the scattering peak significantly distorted the intensities and locations of the previous excitonic peaks [38]. Further, we also observed a broad absorption band centered *ca*. 540 nm. It has been previously assigned as the blue-shifted excitonic peak due to the quantum size effect, indicating that the presence of nanosheets with lateral dimensions less than 50 nm [38].

**Figure 2:**
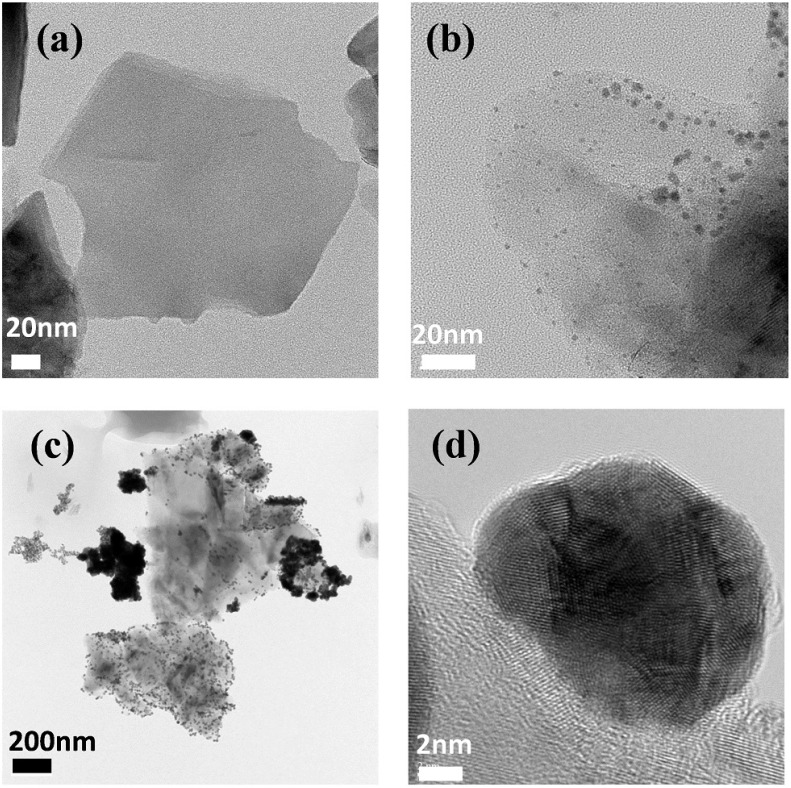
The TEM images of (**a**) MoS_2_ nanosheets; (**b**,**c**) AuNP@MoS_2_. The concentration of the Au ions were (**b**) 0.1 mg·mL^−1^; (**c**) 0.2 mg·mL^−1^; (**d**) High-resolution image of a typical Au NP attached on the MoS_2_ surface.

Meanwhile, Figure 2a shows the transmission electron microscopy (TEM) image of the MoS_2_ nanosheets; the plane size was primarily around 100 nm. In addition, we can also see the coexistence of mono-layer to few-layer of MoS_2_. After MoS_2_ was exfoliated, we further anchored the Au NPs on the MoS_2_ nanosheets through a spontaneous reduction method [37]. An aqueous HAuCl_4_ solution was added to the as-prepared MoS_2_ suspension. Because the Fermi level of MoS_2_ is situated above the reduction potential of AuCl_4_^−^, spontaneous electron transfer from MoS_2_ to Au ions occurred, resulting in the formation of Au NPs on the MoS_2_ surfaces. Figure 2b,c displays the TEM images of the AuNP@MoS_2_ nanocomposites prepared with different concentrations of Au ions. While the concentration of HAuCl_4_ was 0.1 mg·mL^−1^, we clearly found the Au NPs decorated on the MoS_2_ basal planes. The particle size mainly ranged from 2 to 12 nm and the average size was *ca*. 6 nm. The image also shows the preferential edge decoration of the Au NPs, suggesting the reaction preferentially occurred at the highly energetic defect sites [37]. After the concentration of Au ions was increased to 0.2 mg·mL^−1^, more Au NPs were present on the nanosheets. The size of the NPs was slightly increased and the NPs also started to aggregate (Figure 2c). Figure 2d displays the TEM images with a higher magnification. We can see that the Au NP was bonded closely on the MoS_2_ surface. This image suggests that the Au ions were reduced by the nanosheets and the Au NP was directly grown on the MoS_2_ surface rather than in the volume of the solution [40].

The AuNP@MoS_2_ nanocomposites were also characterized using UV–Vis absorption spectroscopy. As shown in Figure 1, after the Au NPs were decorated on the MoS_2_ nanosheets, we observed an intense peak at 535 nm, which is corresponding to the localized surface plasmonic resonance (LSPR) of the Au NPs [17]. As revealed by the previous high-resolution TEM image (Figure 2d), the close contact between the Au NPs and MoS_2_ nanosheets might lead to coupling of their plasmonic resonance. To investigate such possible coupling effect, we reduced AuCl_4_^−^ firstly using sodium citrate and the resulting solution of Au NPs was mixed with the suspension of MoS_2_ nanosheets. From the absorption spectra as displayed in Figure 1, we could clearly observe that the absorption peak of the Au NPs in the “physical” mixing solution was similar to the one of AuNP@MoS_2_ nanocomposite. However, the shapes and intensities of the A and B excitonic peaks were different. Clearly, the two excitonic peaks (as well as the last scattering peak) of the AuNP@MoS_2_ nanocomposite were stronger than those of the physical-mixing solution of Au NPs and MoS_2_ nanosheets. Therefore, we suspect that the plasma electrons in these two components were probably coupled due to their close contact [40].

**Figure 3:**
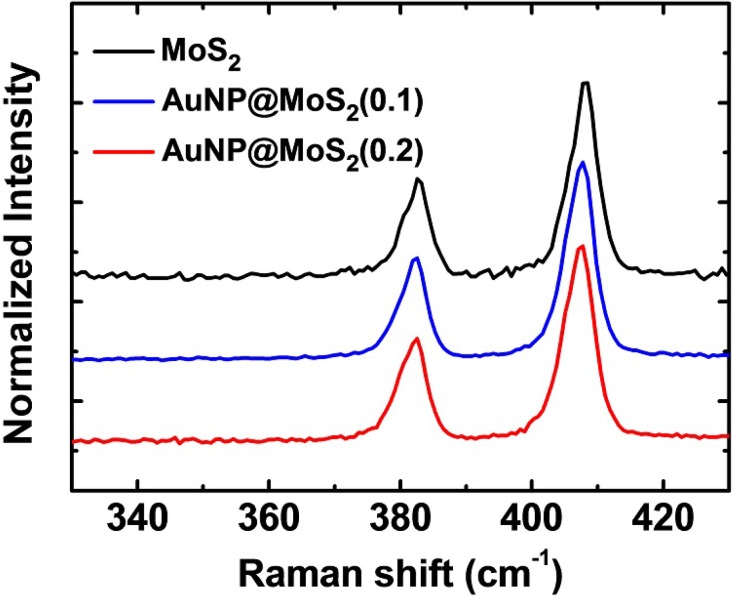
The Raman spectra of AuNP@MoS_2_ nanocompositions. The concentrations of the Au ions were 0.1 and 0.2 mg·mL^−1^, respectively.

Because the van der Waals forces between the atomic layers influence the force constant of the vibrational states, Raman spectroscopy has been proven to be highly useful in probing the structural information of two-dimensional materials [41]. Therefore, we performed Raman spectroscopic measurements and the results were displayed in Figure 3. The MoS_2_ nanosheet featured two main peaks at 382.8 and 407.8 cm^−1^, which are assigned as the E^1^_2g_ and A_1g_ peaks, respectively. They are associated with the in-plane bending (E^1^_2g_) and out-of-plane (A_1g_) vibration modes, respectively. It has been previously shown that the energy difference between these two peaks (Δω) is sensitive to the number of the layers [41]. From Figure 3, we could see that the value of Δω is *ca*. 25 cm^−1^, indicating that the exfoliated MoS_2_ was around 4–5 layers [31,41]. More importantly, the differences between the two peaks were almost unchanged after the MoS_2_ were decorated with Au NPs. Therefore, the Raman spectra suggest that the chemical structure of the nanosheets was not significantly affected by the reduction processes.

### 2.2. Photovoltaic Performance of the Devices Prepared with MoS_2_ Nanocomposites

**Figure 4:**
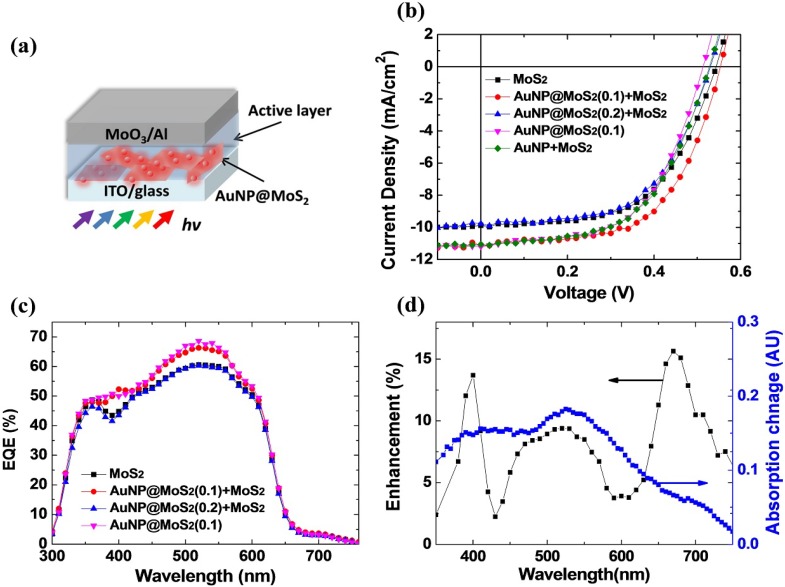
(**a**) The schematic representation of the device structure in this work; (**b**) *J-V* curves of the organic photovoltaic devices (OPVs) prepared with various MoS_2_ nanocomposites; (**c**) Corresponding external quantum efficiency (EQE) curves of these devices; (**d**) The calculated EQE enhancement of the device prepared with the AuNP@MoS_2_ nanocomposites. The change in the absorption spectra after the AuNP@MoS_2_ nanocomposite was also presented for easy comparison.

The as-synthesized MoS_2_ nanocomposites were used as electrode buffer layers in OPVs. Figure 4a displays the device structure incorporating the MoS_2_ nanocomposites. Previous reports have inserted MoS_2_ nanosheets at the anodes [34,35]. Our results, however, indicated that the device using the 2D MoS_2_ as the cathode buffer layers exhibited better device performance, which is consistence with the data reported by Ibrahem *et al.* [31]. The different properties of the 2D MoS_2_ might be due to the different doping levels during the synthesis and device fabrication processes [30,42]. Therefore, we adopted inverted structures to fabricate OPVs with the MoS_2_ nanocomposites. Figure 4b displays the current density–voltage (*J*-*V*) characteristics of the inverted OPVs obtained under illumination with simulated solar light (AM 1.5G); the active layer were P3HT:PCBM blends. Table 1 provides a summary of the electrical properties of the devices in this study. The device prepared with neat MoS_2_ nanosheets as the cathode buffer exhibited a value of *V*_oc_ of 0.55 V, a short-circuit current density (*J*_sc_) of 9.87 mA·cm^−2^, and a fill factor (FF) of 0.56, resulting in a PCE of 3.07%. To investigate the plasmonic effects of these nanocomposites on the performance of OPVs, AuNP@MoS_2_ nanomaterials were also incorporated at the cathode interface. As revealed in Figure 4b, the direct use of the as-synthesized AuNP@MoS_2_ composites led to a decrease value of *V*_oc_ (0.51 V). Although the photocurrent was indeed improved, presumably due to the plasmonic effects, the overall PCE was only slightly improved to 3.19%. We suspected that the density of the Au NPs might be too high, thereby affecting the interfaces between the photoactive polymer blends and the ITO electrodes [18]. Therefore, we further blended neat MoS_2_ nanosheets into the buffer-layer solution to reduce the amount of Au NPs in the devices. As we can see from Figure 4b, the value of *V*_oc_ remained unchanged at 0.55 V and both *J*_sc_ and FF were improved to 11.1 mA·cm^−2^ and 0.59, respectively. The calculated PCE was improved to 3.60%. Moreover, if we used the AuNP@MoS_2_ nanocomposite prepared with higher concentration of Au ions (0.2 mg·mL^−1^), the device performance started to degraded; both *V*_oc_ and *J*_sc_ were reduced, yielding a lower PCE of 2.97%. From the TEM image (Figure 2c), we infer that the aggregated Au NPs probably degraded the cathode interface and the excess Au NPs might also cause strong back scattering. Figure 4b also displays the *J-V* curve of the device prepared with the physical mixing buffer solution as we described in the absorption spectra (Figure 1). The device exhibited a value of *V*_oc_ of 0.53 V, a *J*_sc_ of 11.1 mA·cm^−2^, and a FF of 0.53, resulting in a PCE of 3.21%. The higher photocurrent of the device suggested the present of the plasmonic effects as well [35]. However, the lower value of *V*_oc_, which is probably due to the non-optimized density of the Au NPs, led to the inferior performance. We should note that the best concentration of such device might be different from the condition for the devices using AuNP@MoS_2_ nanocomposite. Further improvement should be still possible after careful optimization of the experimental conditions.

**Table 1:** Electrical characteristics of devices fabricated with MoS_2_ and AuNP@MoS_2_ nanocomposites under various conditions.

Device (concn. of Au ions, mg/mL)	*V*_oc_ (V)	*J*_sc_ (mA·cm^−2^)	FF	PCE (%)
MoS_2_ *^a^*	0.55 ± 0.01	9.87 ± 0.07	0.56 ± 0.01	3.07 ± 0.04
AuNP@MoS_2_(0.10) *^a^*	0.51 ± 0.01	11.2 ± 0.16	0.56 ± 0.01	3.19 ± 0.08
AuNP@MoS_2_(0.10) + MoS_2_ *^a^*	0.55 ± 0.01	11.1 ± 0.11	0.59 ± 0.01	3.60 ± 0.07
AuNP@MoS_2_(0.20) + MoS_2_ *^a^*	0.53 ± 0.01	9.85 ± 0.06	0.57 ± 0.01	2.97 ± 0.05
MoS_2_ *^b^*	0.69 ± 0.01	12.1 ± 0.10	0.52 ± 0.01	4.41 ± 0.06
AuNP@MoS_2_(0.10) + MoS_2_ *^b^*	0.69 ± 0.01	13.4 ± 0.12	0.53 ± 0.01	4.91 ± 0.07

Notes: *^a^*: Photoactive materials: P3HT and PC_61_BM; *^b^*: Photoactive materials: PBDTTT-CT and PC_71_BM.

To further investigate the origin of the device enhancement, we measured the external quantum efficiency (EQE) spectra (Figure 4c). The EQE values improved for the device prepared with the AuNP@MoS_2_ nanocomposite, consistent with the previous *J-V* characteristics. Especially, the efficiencies increased in the wavelength region from 450 to 600 nm, which is consistent with the plasmonic resonance of the Au NPs as shown in Figure 1. The enhancement factor of the plasmonic device compared to the reference MoS_2_ device was further plotted in Figure 4d. The change in the absorption after the AuNP@MoS_2_ nanocomposite was incorporated was also presented for easy comparison. We could also observe both enhancements in EQE values and absorption in the spectral the region ranging from 350 to 450 nm. The results were consistent with previous reports [35]. Although no direct plasmonic resonance of the Au NPs located in this spectral region, the absorption of the devices was still increased possibly due to the scattering effects. Similarly, an enhancement peak could be also observed in the 650–750 nm regions. These spectral features indicated that the scattering scheme also contributed to the device enhancements. Therefore, the EQE spectra indicate that the LSPR of the AuNP@MoS_2_ nanocomposites was responsible for the improved device performance.

**Figure 5:**
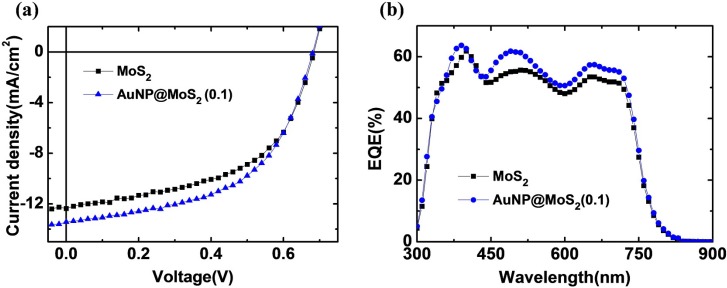
(**a**) *J-V* curves of the OPVs prepared with various MoS_2_ nanocomposites; (**b**) Corresponding EQE curves of these devices. Note that the photoactive material of these OPVs were PBDTTT-CT and PC_71_BM.

To evaluate the potential LSPR effects of the AuNP@MoS_2_ nanocomposites for various OPV applications, we also applied a low-band-gap polymer to fabricate OPVs. For example, a polymer blend consisting of poly{[4,8-bis-(2-ethyl-hexyl-thiophene-5-yl)-benzo[1,2-*b*:4,5-*b*′]dithiophene-2,6-diyl]-alt-[2-(2′-ethyl-hexanoyl)-thieno[3,4-*b*]thiophen-4,6-diyl]} (PBDTTT-CT) and (6,6)-phenyl C_71_-butyric acid methyl ester (PC_71_BM) was used to form the photoactive films; Figure 5 displays the electrical properties of the devices. The reference device prepared with neat MoS_2_ nanosheets exhibited a value of *V*_oc_ of 0.69 V, a value of *J*_sc_ of 12.1 mA·cm^−2^, and a FF of 0.52, yielding a calculated PCE of 4.41%. The PCE value was lower than those reported in the literature [43]. Further improvement might be obtained after some solvent additives, such as 1,8-Diiodooctane, are added in the processing solvent. After the surface of MoS_2_ nanosheets were decorated with Au NPs, the value of *J*_sc_ was improved significantly to 13.4 mA·cm^−2^, while the value of *V*_oc_ remained unchanged, causing the PCE to increase to 4.91%. Figure 5b displays the corresponding EQE spectra. The EQE values also revealed a similar trend with the photocurrent, suggesting that the AuNP@MoS_2_ nanocomposites could indeed improve the light harvesting ability. Notably, the EQE values in the spectral range from 600 to 700 nm were also improved. The origin of the EQE enhancement in this wavelength range is still not clear yet and further investigation is required. However, we suspect that it might be due to the coupling between the plasmonic field of the Au NPs and the MoS_2_ nanosheets as we described previously [36]. Such interesting properties might assist in harvesting the broadband absorption of the solar irradiation.

## 3. Experimental Section

For the synthesis of the MoS_2_ nanocomposites, a 10 mL 1% *w*/*w* suspension of bulk MoS_2_ (Sigma-Aldrich, St. Louis, MO, USA) in DI water was prepared in the first place. Then an aqueous solution of Pluronic P123 (10 wt %; 1.0 mL) (Sigma-Aldrich, St. Louis, MO, USA) was dropped into the MoS_2_ suspension. The feed ratio of Pluronic P123 was 20:70:20 (EO:PO:EO) and its average M_n_ was *ca*. 5.8 kDa. Subsequently, the MoS_2_ blend was sonicated at room temperature for 17 h [36]. After sonication, the as-prepared MoS_2_ solution was washed by toluene to remove P123. Then the MoS_2_ nanosheets were dried through lyophilization. For the preparation of AuNP@MoS_2_ nanocomposites, the dried MoS_2_ nanosheets were dispersed in DI water; the concentration was 0.275 mg·mL^−1^. An aqueous HAuCl_4_ solution (0.1 or 0.2 mg·mL^−1^) (Sigma-Aldrich, St. Louis, MO, USA) was added to the MoS_2_ suspension with a volume ratio of 3:1, respectively. After the spontaneous redox reaction, the resulting suspension was centrifuged, and the residue was washed with toluene and water, respectively. Finally, the nanomaterials were dried through lyophilization.

The devices were prepared on patterned ITO-coated glass substrates. Aqueous solutions of MoS_2_ or AuNP@MoS_2_ (0.07 mg·mL^−1^) were spin-coated onto the ITO substrates and then the sample was baked at 150 °C for 20 min. The photoactive layer, prepared from either a blend of P3HT and PCBM (1:1, *w*/*w*) or a blend of PBDTTT-CT and PC_70_BM (1:1.5, *w*/*w*) in 1,2-dichlorobenzene, was spin-coated onto the MoS_2_ or AuNP@MoS_2_ layers. The photoactive film underwent solvent annealing in a glass Petri dish [44]. Then, the sample was thermally annealed at 110 °C for 15 min. Finally, the device was completed through thermal evaporation of MoO_3_ (3 nm) and Al (100 nm) as the anode. The electrical characteristics of the devices were measured using a Keithley 2400 source-measure unit (Keithley Instruments, Cleveland, OH, USA). A 150-W Thermal Oriel solar simulator (AM 1.5G) was used as the light source druing the meansuremnts. The intensity of the light source was calibrated using a standard Si photodiode equipped with a KG5 filter. The EQE spectra were obtained using a QE measurement system (Enli Technology, Kaohsiung, Taiwan). The absorption spectra were recorded using a UV-Vis-NIR spectrometer (PerkinElmer Lambda 950, Waltham, UK). Raman spectra were acquired using a Horoba high-resolution confocal Raman microscope (HORIBA Scientific, Kyoto, Japan) equipped with a green laser (532 nm) as the light source.

## 4. Conclusions

We have synthesized AuNP@MoS_2_ nanocomposites that could improve the efficiency of OPVs. The AuNP@MoS_2_ nanocomposites were prepared through a simple spontaneous redox reaction between Au ions and MoS_2_ nanosheets. The nanocomposite functioned as the cathode buffer layers and introduced LSPR effects in the devices, thereby resulting in noticeable enhancements in the photocurrent and the PCEs of the OPVs. Moreover, our results reveal the existence of possible coupling of plasmonic resonance between the Au NPs and the MoS_2_ nanosheets, which might be helpful for extending the spectral range of enhanced photon absorption. We anticipate that these results will open up new avenues for improving the performance of OPVs through the exploitation of plasmonic effects in 2D nanomaterials.
